# Ethyl 3-hydr­oxy-13-methyl-4′-phenyl-2′-(3,4,5-trimethoxy­phen­yl)-6,7,8,9,11,12,13,14,15,16-deca­hydro­spiro­[cyclo­penta­[*a*]phenanthrene-16,3′-pyrrolidine]-5′-carboxyl­ate

**DOI:** 10.1107/S1600536808034582

**Published:** 2008-10-31

**Authors:** E. Theboral Sugi Kamala, R. Murugan, S. Nirmala, L. Sudha, S. Sriman Narayanan

**Affiliations:** a Department of Physics, Easwari Engineering College, Ramapuram, Chennai 600 089, India; b Department of Analytical Chemistry, University of Madras, Guindy Campus, Chennai 600025, India; c Department of Physics, SRM University, Ramapuram Campus, Chennai 600 089, India

## Abstract

In the title compound, C_39_H_45_NO_7_,the pyrrolidine ring is connected to an estrone group, a trimeth­oxy benzene and a phenyl ring. The pyrrolidine ring exhibits a twist conformation and the other five-membered ring an envelope conformation. Mol­ecules are linked by N—H⋯O hydrogen bonds, C—H⋯π inter­actions and C—H⋯O hydrogen bonds.

## Related literature

For general background, see: García-Peláez *et al.* (2004 ▶); Holland and Roy (1995 ▶); Obniska *et al.* (2002 ▶); Suzuki *et al.* (1994 ▶). For bond-length data, see: Allen *et al.* (1987 ▶). For puckering parameters, see: Cremer & Pople (1975 ▶). For asymmetry parameters, see: Nardelli (1983 ▶).
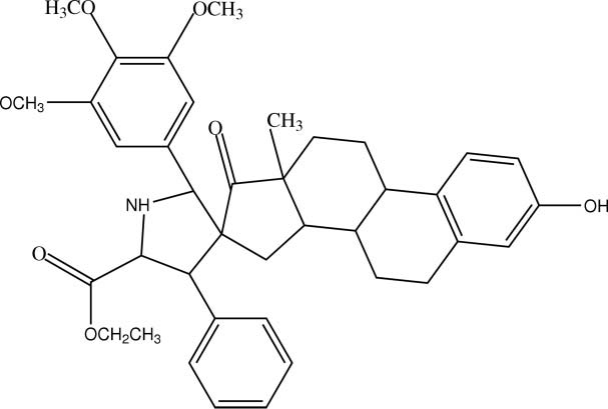


## Experimental

### 

#### Crystal data

C_39_H_45_NO_7_
*M* *_r_* = 639.76
Monoclinic, 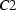

*a* = 26.1776 (6) Å
*b* = 10.3379 (2) Å
*c* = 13.6631 (3) Å
β = 91.1250 (10)°
*V* = 3696.82 (14) Å^3^
*Z* = 4
Mo *K*α radiation
μ = 0.08 mm^−1^
*T* = 293 (2) K
0.30 × 0.30 × 0.25 mm

#### Data collection

Bruker Kappa APEXII diffractometer
Absorption correction: multi-scan (Blessing, 1995 ▶) *T*_min_ = 0.977, *T*_max_ = 0.981
38566 measured reflections
3462 independent reflections
3186 reflections with *I* > 2σ(*I*)
*R*_int_ = 0.027

#### Refinement

*R*[*F* ^2^ > 2σ(*F* ^2^)] = 0.047
*wR*(*F* ^2^) = 0.132
*S* = 1.11
3462 reflections
425 parameters
319 restraints
H-atom parameters constrained
Δρ_max_ = 0.48 e Å^−3^
Δρ_min_ = −0.24 e Å^−3^

### 

Data collection: *APEX2* (Bruker, 2004 ▶); cell refinement: *APEX2* and *SAINT* (Bruker, 2004 ▶); data reduction: *SAINT* and *XPREP* Bruker, 2004 ▶); program(s) used to solve structure: *SHELXS97* (Sheldrick, 2008 ▶); program(s) used to refine structure: *SHELXL97* (Sheldrick, 2008 ▶); molecular graphics: *ORTEP-3* (Farrugia, 1997 ▶); software used to prepare material for publication: *PLATON* (Spek, 2003 ▶).

## Supplementary Material

Crystal structure: contains datablocks I, global. DOI: 10.1107/S1600536808034582/bt2805sup1.cif

Structure factors: contains datablocks I. DOI: 10.1107/S1600536808034582/bt2805Isup2.hkl

Additional supplementary materials:  crystallographic information; 3D view; checkCIF report

## Figures and Tables

**Table 1:** Hydrogen-bond geometry (Å, °)

*D*—H⋯*A*	*D*—H	H⋯*A*	*D*⋯*A*	*D*—H⋯*A*
O2—H2*A*⋯N1^i^	0.82	1.99	2.782 (4)	163
C39—H39*A*⋯O3^ii^	0.96	2.56	3.378 (4)	144
C29—H29*A*⋯*Cg*1^iii^	0.96	3.00	3.820 (4)	144

Symmetry codes: (i) 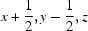; (ii) 
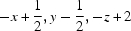
; (iii) 
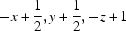
.

**Fig. 1.**
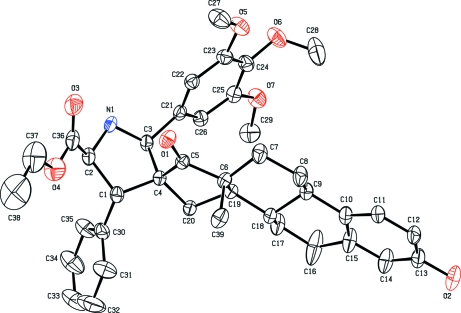
The molecular structure of (I) with 30% probability displacement ellipsoids.

**Fig. 2.**
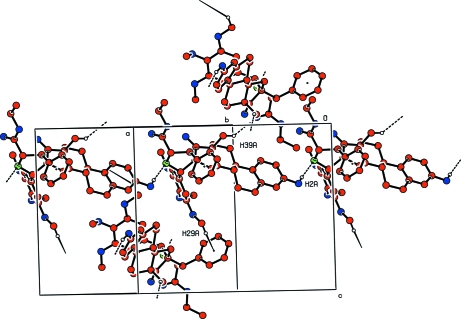
The packing of the molecules viewed down c axis. Hydrogen bonds are indicated by dashed lines and C—H···π interactions are also shown.

## supplementary crystallographic information

### Comment

Estrone is a powerful growth-inducing hormone that is present in milk, mainly in the form of fatty acid esters, at concentrations that promote growth in experimental animals (García-Peláez *et al.*, 2004). Estrone treatment increased proliferation in mammary epithelial cells. In addition, estrone exposure altered cell cycle kinetics (Holland &  Roy, 1995). Derivatives of pyrrolidine are found to have anticonvulsant properties (Obniska *et al.*,2002). Optically active pyrrolidines have been used as intermediates in controlled asymmetric synthesis (Suzuki *et al.*,1994).

Fig 1 shows the *ORTEP*   plot of compound (I). Bond lengths and angles are comparable with other reported values (Allen *et al.*, 1987).

In the molecule the five membered ring N1/C2/C1/C4/C3 exhibits *twist* conformation with assymetry parameters (Nardelli,1983) ΔC_s_ (C4) =15.6 (3), ΔC_2_ (C2) =2.4 (3), and with the puckering parameters (Cremer and Pople,1975) q2 = 0.359 (3) Å, and φ2 = 125.5 (5)°. The ring C4/C5/C6/C19/C20 exhibits *envelope* conformation with envelope on C19 with the assymetry parameters ΔC_s_(C19) = 2.4 (3) and with the puckering parameters q2 = 0.422 (3)Å and φ2 =110.8 (4)°

The six membered ring C9/C10/C15/C16/C17/C18 is perpendicular to the pyrrolidine ring C1/C2/N1/C3/C4 making a dihedral angle of 89.44 (11)° and planar with the five membered ring C4/C5/C6/C19/C20 with a dihedral angle of 2.20 (11).

In the crystal packing, atoms O2 and N1 are involved in intermolecular N—H···O interactions and atom O3 is involved in intermolecular C - H···O interactions. The molecules pack into distinct layers facilitated by C - H···π interactions.

### Experimental

1.0 mole of (*Z*)-16-arylidene estrone (0.78 g) and 1.0 mol of ethyl {[(1E)-(3,4,5-trimethoxyphenyl) methylene] amino} acetate (1.0 g) was stirred in 20 ml of acetonitrile contain AgOAc (0.01 g) and triethylamine (2 ml). The reaction was allowed to stir overnight and the reaction was monitored by TLC. After the completion of reaction, the crude white solid was filtered and then purified by preparative HPLC using water and acetonitrile as eluent. The final pure compound was recrystallized using 2:8 ratio of acetone: hexane.

### Refinement

In the absence of anomalous scatterers Friedel pairs have been merged. H atoms were placed in idealized positions and allowed to ride on their parent atoms, with C–H = 0.93 or 0.96 Å and *U*_iso_(H)= 1.2–1.5*U*_eq_(C).

### Crystal data

**Table:**

C_39_H_45_NO_7_	*F*(000) = 1368
*M**_r_* = 639.76	*D*_x_ = 1.149 Mg m^−^^3^
Monoclinic, *C*2	Mo *K*α radiation, λ = 0.71073 Å
Hall symbol: C 2y	Cell parameters from 38566 reflections
*a* = 26.1776 (6) Å	θ = 1.5–30.7°
*b* = 10.3379 (2) Å	µ = 0.08 mm^−^^1^
*c* = 13.6631 (3) Å	*T* = 293 K
β = 91.125 (1)°	Prism, colourless
*V* = 3696.82 (14) Å^3^	0.30 × 0.30 × 0.25 mm
*Z* = 4	

### Data collection

**Table:**

Bruker KappaAPEXII diffractometer	3462 independent reflections
Radiation source: fine-focus sealed tube	3186 reflections with *I* > 2σ(*I*)
graphite	*R*_int_ = 0.027
ω and φ scans	θ_max_ = 25.0°, θ_min_ = 1.5°
Absorption correction: multi-scan (Blessing, 1995)	*h* = −31→31
*T*_min_ = 0.977, *T*_max_ = 0.981	*k* = −12→12
38566 measured reflections	*l* = −16→15

### Refinement

**Table:**

Refinement on *F*^2^	Secondary atom site location: difference Fourier map
Least-squares matrix: full	Hydrogen site location: inferred from neighbouring sites
*R*[*F*^2^ > 2σ(*F*^2^)] = 0.047	H-atom parameters constrained
*wR*(*F*^2^) = 0.132	*w* = 1/[σ^2^(*F*_o_^2^) + (0.082*P*)^2^ + 1.3827*P*] where *P* = (*F*_o_^2^ + 2*F*_c_^2^)/3
*S* = 1.11	(Δ/σ)_max_ < 0.001
3462 reflections	Δρ_max_ = 0.48 e Å^−^^3^
425 parameters	Δρ_min_ = −0.24 e Å^−^^3^
319 restraints	Extinction correction: SHELXL97 (Sheldrick, 2008), Fc^*^=kFc[1+0.001xFc^2^λ^3^/sin(2θ)]^-1/4^
Primary atom site location: structure-invariant direct methods	Extinction coefficient: 0.0112 (9)

### Special details

**Table:**

Geometry. All e.s.d.'s (except the e.s.d. in the dihedral angle between two l.s. planes) are estimated using the full covariance matrix. The cell e.s.d.'s are taken into account individually in the estimation of e.s.d.'s in distances, angles and torsion angles; correlations between e.s.d.'s in cell parameters are only used when they are defined by crystal symmetry. An approximate (isotropic) treatment of cell e.s.d.'s is used for estimating e.s.d.'s involving l.s. planes.
Refinement. Refinement of *F*^2^ against ALL reflections. The weighted *R*-factor *wR* and goodness of fit *S* are based on *F*^2^, conventional *R*-factors *R* are based on *F*, with *F* set to zero for negative *F*^2^. The threshold expression of *F*^2^ > σ(*F*^2^) is used only for calculating *R*-factors(gt) *etc*. and is not relevant to the choice of reflections for refinement. *R*-factors based on *F*^2^ are statistically about twice as large as those based on *F*, and *R*- factors based on ALL data will be even larger.

### Fractional atomic coordinates and isotropic or equivalent isotropic displacement parameters (Å^2^)

**Table:**

	*x*	*y*	*z*	*U*_iso_*/*U*_eq_	
C1	0.15174 (10)	0.2439 (3)	0.8431 (2)	0.0417 (6)	
H1	0.1599	0.2242	0.9117	0.050*	
C2	0.11604 (10)	0.3642 (3)	0.8400 (2)	0.0460 (7)	
H2	0.0836	0.3374	0.8093	0.055*	
C3	0.17981 (9)	0.3995 (3)	0.7236 (2)	0.0407 (6)	
H3	0.1638	0.3548	0.6676	0.049*	
C4	0.20146 (9)	0.2927 (3)	0.79377 (18)	0.0367 (6)	
C5	0.23722 (10)	0.3500 (3)	0.87263 (18)	0.0380 (6)	
C6	0.28956 (10)	0.2904 (3)	0.86410 (19)	0.0400 (6)	
C7	0.33546 (11)	0.3760 (4)	0.8831 (2)	0.0569 (8)	
H7A	0.3366	0.4019	0.9514	0.068*	
H7B	0.3327	0.4535	0.8433	0.068*	
C8	0.38453 (11)	0.3024 (4)	0.8583 (2)	0.0591 (9)	
H8A	0.4135	0.3602	0.8659	0.071*	
H8B	0.3892	0.2317	0.9044	0.071*	
C9	0.38342 (10)	0.2479 (3)	0.7540 (2)	0.0459 (7)	
H9	0.3827	0.3227	0.7099	0.055*	
C10	0.43051 (10)	0.1699 (3)	0.7281 (2)	0.0491 (7)	
C11	0.47581 (11)	0.1772 (4)	0.7813 (2)	0.0525 (7)	
H11	0.4775	0.2290	0.8369	0.063*	
C12	0.51874 (11)	0.1095 (4)	0.7539 (3)	0.0581 (8)	
H12	0.5489	0.1171	0.7905	0.070*	
C13	0.51690 (11)	0.0307 (4)	0.6726 (2)	0.0568 (8)	
C14	0.47166 (13)	0.0182 (5)	0.6216 (3)	0.0717 (11)	
H14	0.4699	−0.0366	0.5676	0.086*	
C15	0.42859 (12)	0.0850 (5)	0.6485 (2)	0.0669 (10)	
C16	0.37936 (15)	0.0587 (8)	0.5916 (3)	0.118 (3)	
H16A	0.3851	0.0757	0.5228	0.141*	
H16B	0.3711	−0.0323	0.5979	0.141*	
C17	0.33571 (11)	0.1342 (4)	0.6219 (2)	0.0580 (9)	
H17A	0.3049	0.0859	0.6061	0.070*	
H17B	0.3346	0.2135	0.5839	0.070*	
C18	0.33529 (10)	0.1692 (3)	0.7300 (2)	0.0398 (6)	
H18	0.3360	0.0896	0.7688	0.048*	
C19	0.28882 (9)	0.2477 (3)	0.75683 (18)	0.0354 (5)	
H19	0.2904	0.3273	0.7180	0.042*	
C20	0.23490 (9)	0.1939 (3)	0.7407 (2)	0.0391 (6)	
H20A	0.2317	0.1084	0.7692	0.047*	
H20B	0.2260	0.1896	0.6715	0.047*	
C21	0.21851 (9)	0.4925 (3)	0.68252 (19)	0.0395 (6)	
C22	0.23850 (11)	0.5940 (3)	0.7375 (2)	0.0428 (6)	
H22	0.2258	0.6113	0.7993	0.051*	
C23	0.27723 (12)	0.6695 (3)	0.7007 (2)	0.0466 (7)	
C24	0.29617 (11)	0.6431 (3)	0.6073 (2)	0.0482 (7)	
C25	0.27614 (11)	0.5418 (3)	0.55345 (19)	0.0450 (7)	
C26	0.23668 (10)	0.4680 (3)	0.5897 (2)	0.0423 (6)	
H26	0.2224	0.4019	0.5519	0.051*	
C27	0.28307 (18)	0.8008 (5)	0.8430 (3)	0.0772 (11)	
H27A	0.3020	0.8736	0.8682	0.093*	
H27B	0.2883	0.7277	0.8853	0.093*	
H27C	0.2474	0.8218	0.8398	0.093*	
C28	0.38287 (18)	0.6790 (7)	0.5804 (4)	0.1085 (19)	
H28A	0.4057	0.7396	0.5509	0.130*	
H28B	0.3864	0.5960	0.5497	0.130*	
H28C	0.3911	0.6716	0.6490	0.130*	
C29	0.28402 (16)	0.4093 (4)	0.4123 (2)	0.0656 (9)	
H29A	0.3019	0.4074	0.3516	0.079*	
H29B	0.2479	0.4091	0.3991	0.079*	
H29C	0.2931	0.3346	0.4505	0.079*	
C30	0.12845 (12)	0.1248 (3)	0.7966 (2)	0.0509 (7)	
C31	0.1356 (2)	0.0062 (4)	0.8399 (4)	0.0837 (13)	
H31	0.1546	0.0003	0.8979	0.100*	
C32	0.1148 (3)	−0.1060 (5)	0.7983 (5)	0.120 (2)	
H32	0.1199	−0.1855	0.8288	0.143*	
C33	0.0873 (3)	−0.0988 (6)	0.7140 (5)	0.1109 (19)	
H33	0.0732	−0.1733	0.6865	0.133*	
C34	0.0803 (2)	0.0170 (6)	0.6697 (4)	0.0965 (16)	
H34	0.0614	0.0216	0.6113	0.116*	
C35	0.10066 (15)	0.1279 (4)	0.7099 (3)	0.0680 (10)	
H35	0.0956	0.2065	0.6781	0.082*	
C36	0.10410 (11)	0.4229 (4)	0.9382 (3)	0.0603 (9)	
C37	0.0691 (2)	0.3797 (8)	1.0936 (4)	0.120 (2)	
H37A	0.0974	0.4174	1.1306	0.144*	
H37B	0.0430	0.4453	1.0846	0.144*	
C38	0.0492 (3)	0.2730 (11)	1.1447 (4)	0.171 (4)	
H38A	0.0373	0.3011	1.2072	0.205*	
H38B	0.0754	0.2091	1.1541	0.205*	
H38C	0.0213	0.2362	1.1075	0.205*	
C39	0.28908 (13)	0.1760 (4)	0.9367 (2)	0.0575 (8)	
H39A	0.3214	0.1320	0.9354	0.069*	
H39B	0.2623	0.1170	0.9183	0.069*	
H39C	0.2833	0.2079	1.0015	0.069*	
N1	0.13869 (9)	0.4615 (3)	0.7770 (2)	0.0521 (7)	
H1A	0.1295	0.5412	0.7721	0.062*	
O1	0.22398 (8)	0.4254 (2)	0.93491 (14)	0.0518 (5)	
O2	0.55830 (9)	−0.0346 (3)	0.64077 (19)	0.0773 (9)	
H2A	0.5829	−0.0194	0.6771	0.093*	
O3	0.10751 (10)	0.5359 (3)	0.9562 (2)	0.0814 (9)	
O4	0.08664 (12)	0.3347 (3)	0.99822 (19)	0.0842 (9)	
O5	0.30005 (10)	0.7705 (2)	0.74826 (17)	0.0641 (7)	
O6	0.33211 (10)	0.7226 (3)	0.56819 (18)	0.0708 (7)	
O7	0.29758 (9)	0.5228 (3)	0.46488 (15)	0.0590 (6)	

### Atomic displacement parameters (Å^2^)

**Table:**

	*U*^11^	*U*^22^	*U*^33^	*U*^12^	*U*^13^	*U*^23^
C1	0.0373 (13)	0.0503 (15)	0.0380 (13)	0.0004 (12)	0.0077 (10)	0.0014 (12)
C2	0.0314 (13)	0.0572 (18)	0.0494 (15)	−0.0013 (12)	0.0049 (11)	−0.0071 (14)
C3	0.0294 (12)	0.0489 (15)	0.0438 (13)	0.0008 (11)	0.0005 (10)	0.0030 (13)
C4	0.0330 (12)	0.0394 (13)	0.0379 (12)	0.0011 (11)	0.0033 (10)	−0.0006 (12)
C5	0.0386 (13)	0.0412 (14)	0.0344 (12)	0.0029 (12)	0.0039 (10)	0.0009 (12)
C6	0.0368 (13)	0.0463 (15)	0.0368 (13)	0.0070 (12)	−0.0018 (10)	−0.0014 (12)
C7	0.0415 (15)	0.072 (2)	0.0566 (17)	0.0004 (15)	−0.0050 (13)	−0.0224 (17)
C8	0.0362 (14)	0.079 (2)	0.0620 (18)	−0.0012 (15)	−0.0075 (13)	−0.0192 (18)
C9	0.0325 (13)	0.0553 (17)	0.0498 (15)	0.0026 (13)	−0.0011 (11)	0.0011 (14)
C10	0.0327 (13)	0.0632 (18)	0.0513 (16)	0.0037 (13)	0.0038 (11)	0.0072 (15)
C11	0.0336 (14)	0.0558 (17)	0.0679 (19)	−0.0019 (13)	−0.0018 (12)	0.0006 (16)
C12	0.0305 (13)	0.065 (2)	0.078 (2)	0.0023 (14)	−0.0050 (13)	0.0093 (19)
C13	0.0363 (14)	0.073 (2)	0.0610 (19)	0.0101 (15)	0.0103 (13)	0.0167 (18)
C14	0.0452 (17)	0.112 (3)	0.0578 (19)	0.016 (2)	0.0070 (14)	−0.014 (2)
C15	0.0367 (15)	0.112 (3)	0.0526 (17)	0.0153 (18)	0.0035 (13)	−0.010 (2)
C16	0.050 (2)	0.225 (7)	0.078 (3)	0.037 (3)	−0.0085 (19)	−0.071 (4)
C17	0.0374 (14)	0.085 (2)	0.0515 (17)	0.0059 (16)	0.0002 (12)	−0.0226 (17)
C18	0.0317 (12)	0.0443 (14)	0.0435 (14)	0.0052 (11)	0.0028 (10)	−0.0001 (12)
C19	0.0323 (12)	0.0379 (13)	0.0359 (12)	0.0053 (11)	0.0001 (9)	−0.0016 (11)
C20	0.0327 (13)	0.0430 (15)	0.0416 (14)	0.0025 (11)	0.0021 (10)	−0.0044 (12)
C21	0.0314 (12)	0.0442 (15)	0.0427 (14)	0.0064 (11)	−0.0006 (10)	0.0054 (12)
C22	0.0426 (14)	0.0450 (15)	0.0410 (14)	0.0040 (12)	0.0073 (11)	0.0034 (12)
C23	0.0510 (16)	0.0418 (15)	0.0472 (15)	−0.0010 (13)	0.0031 (12)	0.0018 (13)
C24	0.0482 (16)	0.0516 (17)	0.0449 (15)	−0.0057 (13)	0.0066 (12)	0.0081 (13)
C25	0.0442 (15)	0.0551 (17)	0.0357 (13)	0.0044 (13)	0.0026 (11)	0.0085 (13)
C26	0.0401 (13)	0.0475 (16)	0.0391 (14)	0.0019 (12)	−0.0036 (11)	0.0058 (12)
C27	0.099 (3)	0.070 (2)	0.064 (2)	−0.024 (2)	0.017 (2)	−0.016 (2)
C28	0.061 (3)	0.145 (5)	0.120 (4)	−0.030 (3)	0.023 (3)	−0.018 (4)
C29	0.083 (2)	0.069 (2)	0.0449 (17)	0.002 (2)	0.0097 (15)	−0.0035 (16)
C30	0.0479 (16)	0.0509 (17)	0.0545 (18)	−0.0075 (14)	0.0158 (13)	0.0025 (14)
C31	0.105 (3)	0.060 (2)	0.086 (3)	−0.019 (2)	0.006 (2)	0.017 (2)
C32	0.158 (6)	0.055 (3)	0.146 (5)	−0.036 (3)	0.024 (5)	0.013 (3)
C33	0.135 (5)	0.080 (3)	0.118 (4)	−0.057 (3)	0.017 (4)	−0.015 (3)
C34	0.107 (4)	0.099 (4)	0.083 (3)	−0.052 (3)	0.004 (3)	−0.016 (3)
C35	0.075 (2)	0.065 (2)	0.064 (2)	−0.025 (2)	0.0030 (18)	−0.0038 (18)
C36	0.0352 (14)	0.080 (3)	0.066 (2)	0.0036 (16)	0.0065 (13)	−0.022 (2)
C37	0.121 (4)	0.174 (6)	0.066 (3)	0.014 (4)	0.044 (3)	−0.029 (4)
C38	0.201 (7)	0.237 (10)	0.077 (3)	0.035 (8)	0.069 (4)	0.023 (6)
C39	0.0578 (18)	0.075 (2)	0.0403 (15)	0.0201 (17)	0.0034 (13)	0.0109 (16)
N1	0.0324 (11)	0.0493 (14)	0.0749 (18)	0.0119 (11)	0.0116 (11)	0.0090 (13)
O1	0.0530 (11)	0.0588 (13)	0.0436 (10)	0.0108 (10)	0.0032 (9)	−0.0135 (10)
O2	0.0427 (12)	0.116 (2)	0.0739 (15)	0.0270 (14)	0.0088 (11)	0.0065 (16)
O3	0.0622 (15)	0.080 (2)	0.102 (2)	0.0025 (14)	0.0078 (14)	−0.0431 (18)
O4	0.099 (2)	0.096 (2)	0.0591 (14)	0.0074 (17)	0.0375 (14)	−0.0088 (15)
O5	0.0755 (15)	0.0619 (15)	0.0554 (12)	−0.0238 (13)	0.0129 (11)	−0.0080 (12)
O6	0.0726 (16)	0.0733 (17)	0.0673 (15)	−0.0251 (14)	0.0218 (12)	0.0038 (13)
O7	0.0677 (14)	0.0703 (15)	0.0396 (10)	−0.0097 (12)	0.0127 (9)	0.0016 (11)

### Geometric parameters (Å, °)

**Table:**

C1—C30	1.509 (4)	C20—H20B	0.9700
C1—C2	1.555 (4)	C21—C26	1.386 (4)
C1—C4	1.561 (4)	C21—C22	1.387 (4)
C1—H1	0.9800	C22—C23	1.382 (4)
C2—N1	1.458 (4)	C22—H22	0.9300
C2—C36	1.511 (4)	C23—O5	1.362 (4)
C2—H2	0.9800	C23—C24	1.404 (4)
C3—N1	1.460 (4)	C24—O6	1.366 (4)
C3—C21	1.512 (4)	C24—C25	1.378 (4)
C3—C4	1.562 (4)	C25—O7	1.358 (3)
C3—H3	0.9800	C25—C26	1.384 (4)
C4—C5	1.533 (4)	C26—H26	0.9300
C4—C20	1.536 (4)	C27—O5	1.411 (4)
C5—O1	1.210 (3)	C27—H27A	0.9600
C5—C6	1.509 (4)	C27—H27B	0.9600
C6—C7	1.511 (4)	C27—H27C	0.9600
C6—C19	1.530 (4)	C28—O6	1.410 (6)
C6—C39	1.544 (5)	C28—H28A	0.9600
C7—C8	1.537 (5)	C28—H28B	0.9600
C7—H7A	0.9700	C28—H28C	0.9600
C7—H7B	0.9700	C29—O7	1.417 (5)
C8—C9	1.532 (4)	C29—H29A	0.9600
C8—H8A	0.9700	C29—H29B	0.9600
C8—H8B	0.9700	C29—H29C	0.9600
C9—C10	1.521 (4)	C30—C31	1.373 (6)
C9—C18	1.530 (4)	C30—C35	1.378 (5)
C9—H9	0.9800	C31—C32	1.396 (7)
C10—C11	1.380 (4)	C31—H31	0.9300
C10—C15	1.399 (5)	C32—C33	1.348 (9)
C11—C12	1.382 (5)	C32—H32	0.9300
C11—H11	0.9300	C33—C34	1.353 (9)
C12—C13	1.378 (5)	C33—H33	0.9300
C12—H12	0.9300	C34—C35	1.376 (6)
C13—O2	1.356 (4)	C34—H34	0.9300
C13—C14	1.369 (5)	C35—H35	0.9300
C14—C15	1.378 (5)	C36—O3	1.197 (5)
C14—H14	0.9300	C36—O4	1.314 (5)
C15—C16	1.516 (5)	C37—C38	1.411 (12)
C16—C17	1.450 (5)	C37—O4	1.466 (5)
C16—H16A	0.9700	C37—H37A	0.9700
C16—H16B	0.9700	C37—H37B	0.9700
C17—C18	1.522 (4)	C38—H38A	0.9600
C17—H17A	0.9700	C38—H38B	0.9600
C17—H17B	0.9700	C38—H38C	0.9600
C18—C19	1.513 (4)	C39—H39A	0.9600
C18—H18	0.9800	C39—H39B	0.9600
C19—C20	1.529 (4)	C39—H39C	0.9600
C19—H19	0.9800	N1—H1A	0.8600
C20—H20A	0.9700	O2—H2A	0.8200
			
C30—C1—C2	113.8 (2)	C20—C19—H19	106.0
C30—C1—C4	114.6 (2)	C6—C19—H19	106.0
C2—C1—C4	103.7 (2)	C19—C20—C4	102.9 (2)
C30—C1—H1	108.2	C19—C20—H20A	111.2
C2—C1—H1	108.2	C4—C20—H20A	111.2
C4—C1—H1	108.2	C19—C20—H20B	111.2
N1—C2—C36	109.9 (3)	C4—C20—H20B	111.2
N1—C2—C1	108.4 (2)	H20A—C20—H20B	109.1
C36—C2—C1	115.6 (3)	C26—C21—C22	120.1 (3)
N1—C2—H2	107.5	C26—C21—C3	117.8 (3)
C36—C2—H2	107.5	C22—C21—C3	121.9 (2)
C1—C2—H2	107.5	C23—C22—C21	120.1 (3)
N1—C3—C21	114.3 (2)	C23—C22—H22	120.0
N1—C3—C4	105.4 (2)	C21—C22—H22	120.0
C21—C3—C4	116.1 (2)	O5—C23—C22	125.2 (3)
N1—C3—H3	106.8	O5—C23—C24	115.0 (3)
C21—C3—H3	106.8	C22—C23—C24	119.7 (3)
C4—C3—H3	106.8	O6—C24—C25	120.4 (3)
C5—C4—C20	104.1 (2)	O6—C24—C23	119.8 (3)
C5—C4—C1	108.9 (2)	C25—C24—C23	119.7 (3)
C20—C4—C1	118.5 (2)	O7—C25—C24	115.2 (3)
C5—C4—C3	111.5 (2)	O7—C25—C26	124.3 (3)
C20—C4—C3	112.6 (2)	C24—C25—C26	120.5 (3)
C1—C4—C3	101.4 (2)	C25—C26—C21	119.9 (3)
O1—C5—C6	126.2 (2)	C25—C26—H26	120.1
O1—C5—C4	124.4 (2)	C21—C26—H26	120.1
C6—C5—C4	109.3 (2)	O5—C27—H27A	109.5
C5—C6—C7	117.9 (3)	O5—C27—H27B	109.5
C5—C6—C19	101.4 (2)	H27A—C27—H27B	109.5
C7—C6—C19	109.2 (2)	O5—C27—H27C	109.5
C5—C6—C39	104.1 (2)	H27A—C27—H27C	109.5
C7—C6—C39	110.8 (3)	H27B—C27—H27C	109.5
C19—C6—C39	113.2 (3)	O6—C28—H28A	109.5
C6—C7—C8	109.7 (3)	O6—C28—H28B	109.5
C6—C7—H7A	109.7	H28A—C28—H28B	109.5
C8—C7—H7A	109.7	O6—C28—H28C	109.5
C6—C7—H7B	109.7	H28A—C28—H28C	109.5
C8—C7—H7B	109.7	H28B—C28—H28C	109.5
H7A—C7—H7B	108.2	O7—C29—H29A	109.5
C9—C8—C7	112.7 (2)	O7—C29—H29B	109.5
C9—C8—H8A	109.0	H29A—C29—H29B	109.5
C7—C8—H8A	109.0	O7—C29—H29C	109.5
C9—C8—H8B	109.0	H29A—C29—H29C	109.5
C7—C8—H8B	109.0	H29B—C29—H29C	109.5
H8A—C8—H8B	107.8	C31—C30—C35	117.1 (4)
C10—C9—C18	109.6 (3)	C31—C30—C1	119.9 (3)
C10—C9—C8	114.2 (2)	C35—C30—C1	123.0 (3)
C18—C9—C8	113.3 (2)	C30—C31—C32	121.2 (5)
C10—C9—H9	106.4	C30—C31—H31	119.4
C18—C9—H9	106.4	C32—C31—H31	119.4
C8—C9—H9	106.4	C33—C32—C31	119.9 (5)
C11—C10—C15	117.4 (3)	C33—C32—H32	120.0
C11—C10—C9	122.8 (3)	C31—C32—H32	120.0
C15—C10—C9	119.8 (2)	C32—C33—C34	119.8 (5)
C10—C11—C12	121.7 (3)	C32—C33—H33	120.1
C10—C11—H11	119.2	C34—C33—H33	120.1
C12—C11—H11	119.2	C33—C34—C35	120.7 (5)
C13—C12—C11	120.1 (3)	C33—C34—H34	119.7
C13—C12—H12	119.9	C35—C34—H34	119.7
C11—C12—H12	119.9	C34—C35—C30	121.2 (4)
O2—C13—C14	118.6 (3)	C34—C35—H35	119.4
O2—C13—C12	122.5 (3)	C30—C35—H35	119.4
C14—C13—C12	118.9 (3)	O3—C36—O4	125.1 (4)
C13—C14—C15	121.4 (4)	O3—C36—C2	124.0 (4)
C13—C14—H14	119.3	O4—C36—C2	110.8 (3)
C15—C14—H14	119.3	C38—C37—O4	108.5 (6)
C14—C15—C10	120.3 (3)	C38—C37—H37A	110.0
C14—C15—C16	117.7 (4)	O4—C37—H37A	110.0
C10—C15—C16	121.9 (3)	C38—C37—H37B	110.0
C17—C16—C15	115.1 (4)	O4—C37—H37B	110.0
C17—C16—H16A	108.5	H37A—C37—H37B	108.4
C15—C16—H16A	108.5	C37—C38—H38A	109.5
C17—C16—H16B	108.5	C37—C38—H38B	109.5
C15—C16—H16B	108.5	H38A—C38—H38B	109.5
H16A—C16—H16B	107.5	C37—C38—H38C	109.5
C16—C17—C18	115.2 (3)	H38A—C38—H38C	109.5
C16—C17—H17A	108.5	H38B—C38—H38C	109.5
C18—C17—H17A	108.5	C6—C39—H39A	109.5
C16—C17—H17B	108.5	C6—C39—H39B	109.5
C18—C17—H17B	108.5	H39A—C39—H39B	109.5
H17A—C17—H17B	107.5	C6—C39—H39C	109.5
C19—C18—C17	112.6 (2)	H39A—C39—H39C	109.5
C19—C18—C9	109.0 (2)	H39B—C39—H39C	109.5
C17—C18—C9	108.3 (2)	C2—N1—C3	107.7 (2)
C19—C18—H18	109.0	C2—N1—H1A	126.1
C17—C18—H18	109.0	C3—N1—H1A	126.1
C9—C18—H18	109.0	C13—O2—H2A	109.5
C18—C19—C20	120.9 (2)	C36—O4—C37	117.0 (4)
C18—C19—C6	113.0 (2)	C23—O5—C27	117.7 (3)
C20—C19—C6	103.7 (2)	C24—O6—C28	114.6 (4)
C18—C19—H19	106.0	C25—O7—C29	117.9 (3)
			
C30—C1—C2—N1	−114.0 (3)	C17—C18—C19—C6	−176.6 (3)
C4—C1—C2—N1	11.1 (3)	C9—C18—C19—C6	−56.4 (3)
C30—C1—C2—C36	122.1 (3)	C5—C6—C19—C18	−173.3 (2)
C4—C1—C2—C36	−112.8 (3)	C7—C6—C19—C18	61.6 (3)
C30—C1—C4—C5	−145.9 (3)	C39—C6—C19—C18	−62.3 (3)
C2—C1—C4—C5	89.5 (3)	C5—C6—C19—C20	−40.5 (3)
C30—C1—C4—C20	−27.4 (3)	C7—C6—C19—C20	−165.6 (2)
C2—C1—C4—C20	−151.9 (2)	C39—C6—C19—C20	70.4 (3)
C30—C1—C4—C3	96.3 (3)	C18—C19—C20—C4	170.4 (2)
C2—C1—C4—C3	−28.3 (3)	C6—C19—C20—C4	42.3 (3)
N1—C3—C4—C5	−78.9 (3)	C5—C4—C20—C19	−26.6 (3)
C21—C3—C4—C5	48.7 (3)	C1—C4—C20—C19	−147.7 (2)
N1—C3—C4—C20	164.5 (2)	C3—C4—C20—C19	94.4 (2)
C21—C3—C4—C20	−67.8 (3)	N1—C3—C21—C26	−139.7 (3)
N1—C3—C4—C1	36.9 (3)	C4—C3—C21—C26	97.2 (3)
C21—C3—C4—C1	164.6 (2)	N1—C3—C21—C22	45.1 (3)
C20—C4—C5—O1	−174.4 (3)	C4—C3—C21—C22	−78.0 (3)
C1—C4—C5—O1	−47.1 (4)	C26—C21—C22—C23	−1.1 (4)
C3—C4—C5—O1	64.0 (3)	C3—C21—C22—C23	174.0 (3)
C20—C4—C5—C6	1.6 (3)	C21—C22—C23—O5	−179.3 (3)
C1—C4—C5—C6	128.9 (2)	C21—C22—C23—C24	0.1 (4)
C3—C4—C5—C6	−120.0 (2)	O5—C23—C24—O6	−4.7 (4)
O1—C5—C6—C7	−41.2 (4)	C22—C23—C24—O6	175.8 (3)
C4—C5—C6—C7	142.9 (3)	O5—C23—C24—C25	179.2 (3)
O1—C5—C6—C19	−160.2 (3)	C22—C23—C24—C25	−0.3 (4)
C4—C5—C6—C19	23.9 (3)	O6—C24—C25—O7	5.0 (4)
O1—C5—C6—C39	82.0 (4)	C23—C24—C25—O7	−178.9 (3)
C4—C5—C6—C39	−93.9 (3)	O6—C24—C25—C26	−174.5 (3)
C5—C6—C7—C8	−173.0 (3)	C23—C24—C25—C26	1.6 (4)
C19—C6—C7—C8	−58.1 (3)	O7—C25—C26—C21	177.9 (3)
C39—C6—C7—C8	67.3 (3)	C24—C25—C26—C21	−2.6 (4)
C6—C7—C8—C9	54.4 (4)	C22—C21—C26—C25	2.4 (4)
C7—C8—C9—C10	−177.8 (3)	C3—C21—C26—C25	−172.9 (2)
C7—C8—C9—C18	−51.3 (4)	C2—C1—C30—C31	−139.2 (4)
C18—C9—C10—C11	−145.8 (3)	C4—C1—C30—C31	101.7 (4)
C8—C9—C10—C11	−17.4 (5)	C2—C1—C30—C35	42.2 (4)
C18—C9—C10—C15	33.1 (4)	C4—C1—C30—C35	−76.8 (4)
C8—C9—C10—C15	161.5 (3)	C35—C30—C31—C32	−1.0 (7)
C15—C10—C11—C12	3.8 (5)	C1—C30—C31—C32	−179.6 (5)
C9—C10—C11—C12	−177.2 (3)	C30—C31—C32—C33	0.2 (10)
C10—C11—C12—C13	−1.0 (5)	C31—C32—C33—C34	0.4 (11)
C11—C12—C13—O2	177.8 (3)	C32—C33—C34—C35	−0.3 (10)
C11—C12—C13—C14	−1.8 (6)	C33—C34—C35—C30	−0.4 (8)
O2—C13—C14—C15	−178.0 (4)	C31—C30—C35—C34	1.1 (6)
C12—C13—C14—C15	1.6 (6)	C1—C30—C35—C34	179.6 (4)
C13—C14—C15—C10	1.3 (7)	N1—C2—C36—O3	7.9 (4)
C13—C14—C15—C16	−176.3 (5)	C1—C2—C36—O3	131.0 (4)
C11—C10—C15—C14	−4.0 (6)	N1—C2—C36—O4	−176.0 (3)
C9—C10—C15—C14	177.0 (4)	C1—C2—C36—O4	−52.9 (4)
C11—C10—C15—C16	173.5 (5)	C36—C2—N1—C3	139.8 (2)
C9—C10—C15—C16	−5.5 (6)	C1—C2—N1—C3	12.5 (3)
C14—C15—C16—C17	−178.6 (5)	C21—C3—N1—C2	−160.0 (2)
C10—C15—C16—C17	3.8 (8)	C4—C3—N1—C2	−31.3 (3)
C15—C16—C17—C18	−31.5 (7)	O3—C36—O4—C37	1.3 (6)
C16—C17—C18—C19	−179.9 (4)	C2—C36—O4—C37	−174.8 (4)
C16—C17—C18—C9	59.5 (5)	C38—C37—O4—C36	176.6 (5)
C10—C9—C18—C19	179.6 (2)	C22—C23—O5—C27	0.2 (5)
C8—C9—C18—C19	50.7 (3)	C24—C23—O5—C27	−179.3 (3)
C10—C9—C18—C17	−57.6 (3)	C25—C24—O6—C28	−86.9 (4)
C8—C9—C18—C17	173.5 (3)	C23—C24—O6—C28	97.0 (4)
C17—C18—C19—C20	59.7 (4)	C24—C25—O7—C29	170.9 (3)
C9—C18—C19—C20	179.8 (2)	C26—C25—O7—C29	−9.6 (4)

### Hydrogen-bond geometry (Å, °)

**Table:**

*D*—H···*A*	*D*—H	H···*A*	*D*···*A*	*D*—H···*A*
O2—H2A···N1^i^	0.82	1.99	2.782 (4)	163
C39—H39A···O3^ii^	0.96	2.56	3.378 (4)	144
C29—H29A···Cg1^iii^	0.96	3.00	3.820 (4)	144
C1—H1···O4	0.98	2.54	2.901 (4)	101

Symmetry codes: (i) *x*+1/2, *y*−1/2, *z*; (ii) −*x*+1/2, *y*−1/2, −*z*+2; (iii) −*x*+1/2, *y*+1/2, −*z*+1.
